# C282Y-HFE Gene Variant Affects Cholesterol Metabolism in Human Neuroblastoma Cells

**DOI:** 10.1371/journal.pone.0088724

**Published:** 2014-02-12

**Authors:** Fatima Ali-Rahmani, Michael A. Huang, C.-L. Schengrund, James R. Connor, Sang Y. Lee

**Affiliations:** 1 Department of Neurosurgery, The Pennsylvania State University College of Medicine, Penn State Hershey Cancer Institute, Penn State M.S. Hershey Medical Center, Hershey, Pennsylvania, United States of America; 2 Division of Pediatric Hematology/Oncology, The Pennsylvania State University College of Medicine, Penn State Hershey Cancer Institute, Penn State M.S. Hershey Medical Center, Hershey, Pennsylvania, United States of America; 3 Department of Biochemistry and Molecular Biology, The Pennsylvania State University College of Medicine, Penn State Hershey Cancer Institute, Penn State M.S. Hershey Medical Center, Hershey, Pennsylvania, United States of America; University of Florida, United States of America

## Abstract

Although disruptions in the maintenance of iron and cholesterol metabolism have been implicated in several cancers, the association between variants in the HFE gene that is associated with cellular iron uptake and cholesterol metabolism has not been studied. The C282Y-HFE variant is a risk factor for different cancers, is known to affect sphingolipid metabolism, and to result in increased cellular iron uptake. The effect of this variant on cholesterol metabolism and its possible relevance to cancer phenotype was investigated using wild type (WT) and C282Y-HFE transfected human neuroblastoma SH-SY5Y cells. Expression of C282Y-HFE in SH-SY5Y cells resulted in a significant increase in total cholesterol as well as increased transcription of a number of genes involved in its metabolism compared to cells expressing WT-HFE. The marked increase in expression of *NPC1L1* relative to that of most other genes, was accompanied by a significant increase in expression of NPC1, a protein that functions in cholesterol uptake by cells. Because inhibitors of cholesterol metabolism have been proposed to be beneficial for treating certain cancers, their effect on the viability of C282Y-HFE neuroblastoma cells was ascertained. C282Y-HFE cells were significantly more sensitive than WT-HFE cells to U18666A, an inhibitor of desmosterol Δ24-reductase the enzyme catalyzing the last step in cholesterol biosynthesis. This was not seen for simvastatin, ezetimibe, or a sphingosine kinase inhibitor. These studies indicate that cancers presenting in carriers of the C282Y-HFE allele might be responsive to treatment designed to selectively reduce cholesterol content in their tumor cells.

## Introduction

Cellular iron uptake is regulated by a number of proteins including HFE (high iron) that when mutated are associated with increased cellular iron uptake and a host of intracellular changes [1]. The wild type (WT)-HFE protein interacts with the transferrin receptor (TfR) to limit uptake of iron-loaded transferrin into the cell. In contrast to plasma membrane-associated WT-HFE, the C282Y-HFE mutant protein is retained in the endoplasmic reticulum (ER) where it does not interact with TfR thus failing to limit iron uptake [2], [3]. A number of studies have shown that C282Y-HFE is associated with accumulation of iron at both the cellular and tissue level [4], [5]. The presence of lower TfR levels in C282Y-HFE cells is an indicator of iron-overload as the cells try to limit further iron uptake by reducing TfR expression [5].

Because of the association of the C282Y-HFE mutation with cellular iron-overload it is logical to propose that the C282Y-HFE gene may be linked to diseases in which iron dysregulation occurs, such as cancer. Results of assessment studies of C282Y-HFE as a risk factor for different types of cancer bore out that supposition. Carriers of C282Y-HFE were found to have an increased risk of breast, ovarian, colorectal, and prostate cancer relative to WT-HFE controls [6]–[9]. Considering that the prevalence of C282Y-HFE homozygosity varies between 3% and 12% in Caucasians [10], and that there is a lack of knowledge about its prevalence in different cancer types, a large number of people may be at an increased risk for either cancer or ineffective therapeutic strategies. The mechanism whereby C282Y-HFE influences cancer incidence is unknown.

Although how C282Y-HFE functions as a risk factor for cancer is unknown, evidence from cell culture studies indicates that it affects key cellular mechanisms. For example, expression of C282Y-HFE in human neuroblastoma cells resulted in increases in intracellular iron, lipid peroxidation, and cell proliferation [5]. In addition, cells expressing C282Y-HFE are resistant to the differentiation agent retinoic acid and to several chemotherapeutic drugs (e.g., doxorubicin) [11]. It is likely that the mechanism of this resistance involves induction of expression of cyclin-dependent kinase inhibitor, p16INK4A [11]. C282Y-HFE expressing cells also exhibit significantly higher expression of sphingosine kinase 1, its pro-survival metabolite sphingosine 1 phosphate, and increased cell proliferation [12]. An epidemiological study found that carriers of the C282Y-HFE mutation had alterations in their serum lipid profile [13], [14]. Though, these studies suggest a link between iron and lipid metabolism, knowledge in this area is limited. So far, there have been no reports describing the effects of C282Y-HFE on cellular cholesterol metabolism. Therefore, in this study we explored this connection using human neuroblastoma SH-SY5Y cell lines stably expressing either WT- or the C282Y-HFE variant because the untransfected cells lack detectable endogenous HFE [5]. They were also selected because of the hypersensitivity of the cell to disruption of lipid rafts [15].

A motivation for pursuing a link between C282Y-HFE and cholesterol metabolism is that unlike WT-HFE, C282Y-HFE does not associate with lipid rafts [12] and because cholesterol is a major lipid component of rafts changes in their protein composition might affect its metabolism. In terms of cell function, lipid rafts provide a platform for protein-protein interactions and play an important role in signal transduction. Alterations in the composition of lipid rafts can have significant effects on cell behavior. The fact that C282Y-HFE is not found in lipid rafts, means that its interaction with other raft-resident proteins is disrupted. Based on our previous finding that metabolism of sphingolipids, a class of lipids also enriched in lipid rafts, was altered in cells expressing C282Y-HFE, we investigated the effect of its expression on cholesterol. We hypothesized that the expression of C282Y-HFE would result in disruption of cholesterol metabolism. To test this hypothesis, we determined the total cholesterol content and monitored expression of genes involved in cholesterol metabolism in C282Y-HFE expressing human neuroblastoma cells. We also determined whether inhibition of cholesterol synthesis/uptake/transport and of sphingosine kinase affected survival of C282Y-HFE cells.

## Materials and Methods

### Materials

Dulbecco's Modified Eagle’s Medium/Nutrient Mixture F-12 (DMEM/F12), fetal bovine serum (FBS) and other cell culture ingredients were purchased from Life Technologies (Grand Island, NY). All PCR Array ingredients were supplied by SABiosciences (Frederick, MD). Ezetimibe was obtained from Selleckchem Co. (Houston, TX) and sphingosine kinase inhibitor (SKI) from EMD Millipore (Billerica, MA). Stock solutions of all test compounds were prepared in 100% DMSO. All other chemicals including simvastatin and U18666A were purchased from Sigma Co. (St. Louis, MO).

### Cell culture, treatment, and cell survival

Human neuroblastoma SH-SY5Y cells, stably transfected to express either FLAG-tagged WT- or the C282Y-HFE alleles [5], were cultured in DMEM/F12 medium containing geneticin (200 µg/mL), 10% FBS, 1% antibiotics (Penn-Strep), 1% non-essential amino acids, and grown at 37°C in an atmosphere of 5% CO_2_/95% air. Because the WT- and C282Y-HFE stably transfected SH-SY5Y cells differed in morphology and in proliferation rate 2.4–3×10^4^ WT- and 0.8–1.5×10^4^ C282Y-HFE SH-SY5Y cells were seeded per well in a 96 well plate and allowed to grow for 1–2 days in DMEM/F12 cell culture medium (10% FBS). This resulted in a relatively equivalent cell confluence at the time cells were washed with Hank’s buffer prior to treating with different concentrations of either simvastatin, ezetimibe, U18666A, or SKI in DMEM/F12 medium containing 1% FBS. Cell survival was measured after 48 hr using the MTS assay according to the manufacturer’s protocol (Promega).

### Cholesterol analyses

Lipids were extracted from equal numbers of cells (10^6^) of each cell type using chloroform (CHCl_3_):isopropanol:Triton X-100 (7:11:0.1). The chloroform layer (bottom) was collected, dried, and extracted material suspended in the cholesterol reaction buffer provided in the Biovision cholesterol assay kit (Mountain View, CA). Cholesterol content was measured using the cholesterol quantification kit according to the manufacturer’s (Biovision) directions. Protein content was determined in an aliquot of each sample of cells using the DC Protein assay (BioRad) prior to cholesterol analysis. Cholesterol content was normalized to protein content and data are presented as percent of WT-HFE control.

### Immunocytochemistry

For a qualitative analysis of cellular cholesterol, cells were stained with filipin, which is known to bind cholesterol [13]. Briefly, 10^5^ cells were seeded per well on collagen coated 4-well chamber slides and maintained as described previously. When cells were confluent, they were washed with phosphate buffered saline (PBS) prior to fixation in 4% paraformaldehyde in PBS (30 min, 4°C). After washing with PBS, cells were stained with filipin (50 µg/ml) for 5 min in the dark. Excess filipin was removed by washing the cells three times in PBS, and the cells visualized by fluorescent microscopy using a Nikon Eclipse 80i microscope that was connected to a Nikon Digital Sight camera. Images were captured using Nikon’s NIS-Elements Br Software version 3.00.

### Gene expression analysis

Total RNA was isolated from SH-SY5Y cells expressing either WT- or C282Y-HFE using the RNeasy Mini Kit (Qiagen, Valencia, CA) according to the manufacturer’s instructions. Recovered RNA was quantified by its absorbance at 260 nm. cDNA was prepared by reverse transcription of isolated RNA (1 µg) using the RT^2^ First Strand Kit (SABiosciences). For qRT-PCR reactions, ∼4ng of cDNA samples were added to a RT^2^ Profiler™ PCR Array (SABiosciences) specific for Human Lipoprotein Signaling & Cholesterol Metabolism (cat # PAHS-080Z). The reactions were carried out according to directions provided with the RT^2^ SYBR^®^ Green/ROX™ qPCR Master Mix Kit (SABiosciences, Frederick, MD). Gene expression of each of the 96 genes was monitored using an ABI 7800 (Applied Biosystems, USA). Data were normalized to the expression of mRNA for actin, GAPDH, and ribosomal protein L13A. This experiment was three times using three different cell preparations. Data were analyzed using online GEAnalysis Suite software (SABiosciences). Any variations among replicates were adjusted by the software and fold changes in gene expression that are consistent between replicates are shown.

### Protein expression

Cells were plated in a 6-well dish and grown to ∼70% confluence. They were then lysed in RIPA buffer (Sigma) containing a protease inhibitor cocktail. After measuring protein concentration using the Pierce BCA protein assay (Pierce, Rockford, IL), equal amounts (50 µg) of WT- and C282Y-HFE cell lysate protein were loaded on a 10% pre-cast polyacrylamide gel (BioRad). The proteins were separated by electrophoresis, transferred onto a PVDF membrane and probed with the following primary antibodies: CXCL16 and APOE (Abcam), NPC1L1 (Santa Cruz), HMGCoAR (Biovision), and β-Actin (Sigma) which was used as a loading control. Anti-mouse or anti-rabbit secondary antibodies were purchased from GE healthcare. Bands were visualized with SuperSignal West Pico or West Femto reagent (Pierce) using the Fuji image analyzer LAS-3000 (Fuji film). Protein expression was normalized to actin and presented as a percentage of WT control.

### Statistical analysis

Data are shown as percentage of the vehicle-treated control or WT-HFE control cells. Each experiment was repeated at least three times with 3–8 replicates. Within each concentration, the cell viability between two groups was compared using two-sample Student's T-test. Data are displayed as mean ± standard error of the mean (SEM). Some error bars are too small to be seen. Differences among means are considered statistically significant when the p-value was less than 0.05.

## Results

### Effect of C282Y-HFE on total cholesterol content in SH-SY5Y cells

**Figure 1:**
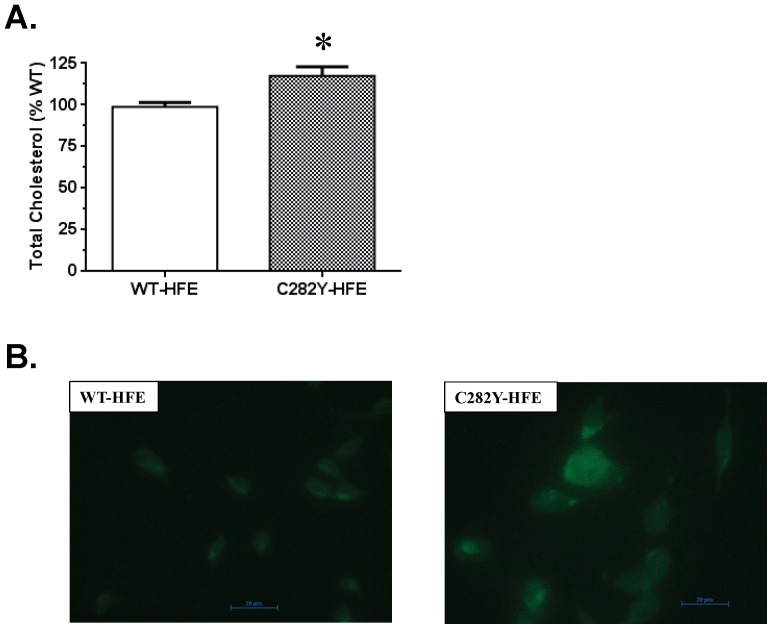
Cholesterol content in C282Y-HFE and WT-HFE stably transfected SH-SY5Y cells. (A) Quantification of total cholesterol in SH-SY5Y cells expressing WT- or C282Y-HFE. Lipids were extracted from 10^6^ cells and cholesterol content determined using a cholesterol quantification kit (Biovision). The cholesterol content in C282Y- HFE cells is indicated relative to that in WT-HFE cells. Data are displayed as means ± SEM. The asterisk (*) indicates a significant (p<0.05, n = 3) increase in cholesterol in C282Y-HFE cells relative to WT-HFE transfected controls. (B) Filipin staining of cholesterol. Cells (10^6^ cells/sample) were cultured in a slide dish for 3 days prior to staining with 1% filipin and visualization using a Nikon Eclipse 80i microscope. Size bars indicate 20 µm.

Biochemical assessment of cholesterol content indicated that C282Y-HFE expressing cells had ∼18% more cholesterol than WT-HFE cells (**Figure 1A**). Qualitative results obtained by monitoring cell staining with filipin, a dye that binds cholesterol, also indicated the presence of more cholesterol in C282Y-HFE cells than in those expressing WT-HFE (**Figure 1B**).

### Effect of C282Y-HFE on expression of genes and proteins affecting cholesterol metabolism

The expression of a number of genes involved in cholesterol metabolism was altered in C282Y-HFE cells relative to WT-HFE cells (**Table 1**). Genes with a 4-fold or greater change in expression were considered to be significantly altered. The two genes whose expression was most significantly increased were NPC1L1 and CXCL16 (>300-fold). These genes encode the proteins Niemann-Pick disease, type C1 gene-like 1, and chemokine ligand 16. Genes with greater than 100-fold up-regulation were those encoding apolipoprotein D (*APOD*), collectin sub-family member 12 (*COLEC12*), and low density lipoprotein-related protein 1B (*LRP1B*). Only one gene, cytochrome P450, family 7, subfamily B, polypeptide 1(*CYP7B1*) was decreased in expression (98-fold) in C282Y-HFE cells compared to WT-HFE cells.

**Table 1:** Effect of C282Y-HFE on the expression of genes involved in lipoprotein signaling and cholesterol metabolism in human neuroblastoma SH-SY5Y cell lines.

*Gene* (Gene Symbol)	Description	Fold changes (C282Y/WT)
*APOA2* (APOA2)	Apolipoprotein A-II	21.4
*APOD* (APOD)	Apolipoprotein D	161.0
*AD2/LPG* (APOE)	Apolipoprotein E	70.6
*Apo-F/DKFZp781G18150* (APOF)	Apolipoprotein F	7.1
*APOL-II* (APOL2)	Apolipoprotein L, 2	6.3
*BAL/BSDL* (CEL)	Carboxyl ester lipase (bile salt-stimulated lipase)	5.0
*HDLCQ10* (CETP)	Cholesteryl ester transfer protein, plasma	8.5
*CLP1/NSR2* (COLEC12)	Collectin sub-family member 12	177.0
*CXCLG16/SR-PSOX* (CXCL16)	Chemokine (C-X-C motif) ligand 16	305.7
*CYP39A1* (CYP39A1)	Cytochrome P450, family 39, subfamily A, polypeptide 1	28.8
*ELA3* (ELA3)	Elastase 3A, pancreatic	38.5
*HMGCS2* (HMGCS2)	3-hydroxy-3-methylglutaryl-Coenzyme A synthase 2 (mitochondrial)	4.0
*BSF1/IL-4* (IL4)	Interleukin 4	7.8
*CL-6* (INSIG1)	Insulin induced gene 1	5.9
*ARH/ARH1* (LDLRAP1)	Low density lipoprotein receptor adaptor protein 1	25.4
*LRP-DIT/LRPDIT* (LRP1B)	Low density lipoprotein-related protein 1B (deleted in tumors)	198.2
*A2MRAP/A2RAP* (LRPAP1)	Low density lipoprotein receptor-related protein associated protein 1	5.2
*NPC1L1* (NPC1L1)	NPC1 (Niemann-Pick disease, type C1, gene)-like 1	332.6
*SHP/SHP1* (NR0B2)	Nuclear receptor subfamily 0, group B, member 2	30.3
*FH/HCHOLA3* (PCSK9)	Proprotein convertase subtilisin/kexin type 9	66.5
*ACACT/ACAT* (SOAT1)	Sterol O-acyltransferase 1	4.1
*CLEVER-1/FEEL-1* (STAB1)	Stabilin 1	8.0
*DKFZp434N127* (ZMYND15)	Zinc finger, MYND-type containing 15	19.9
*CP7B* (CYP7B1)	Cytochrome P450, family 7, subfamily B, polypeptide 1	-98.1

Pools of total RNA from WT- or C282Y-HFE stably transfected cells were analyzed using the GEArray human target specific gene array (Lipoprotein Signaling & Cholesterol Metabolism). Relative changes in mRNA levels between C282Y cells and WT cells are expressed as fold increase (positive number) or fold decrease (negative number). The average changes in expression (> 4 fold) are shown.

**Figure 2:**
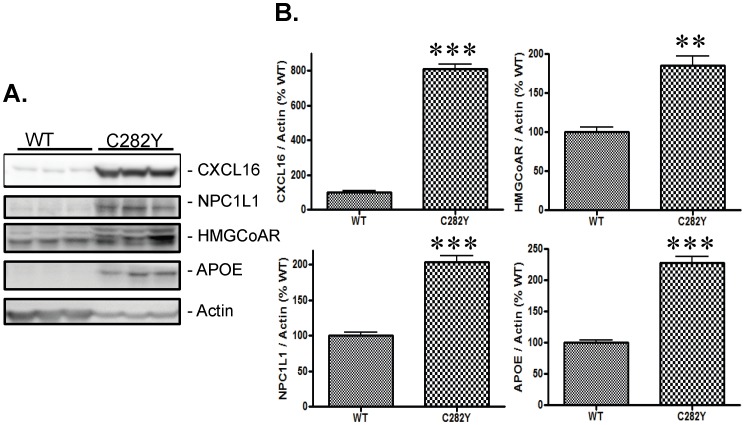
Effect of the C282Y-HFE mutation on expression of proteins involved in cholesterol metabolism in stably transfected SH-SY5Y cells. (A) Protein expression of three of the genes overexpressed in C282Y-HFE cells plus HMGCoA reductase. WT- and C282Y-HFE cells were harvested when ∼70% confluent and protein expression ascertained by Western blotting. (B) Relative expression of proteins in cells expressing either WT- or C282Y-HFE. Data are displayed as means ± SEM. The double asterisk (**) and triple asterisk (***) indicate significance (**p<0.01, n = 3; ***p<0.001, n = 3) of expression in C282Y-HFE cells relative to WT-HFE controls.

Western blot analyses of the protein expression for a number of genes that were significantly altered in gene array analyses showed a significant induction of CXCL16 (p<0.001) (a scavenger receptor on macrophages, inducer of chemotactic response and calcium mobilization) and NPC1L1 (p<0.001) (Niemann-Pick C1-like protein, has a major role in cholesterol uptake) in C282Y-HFE cells relative to WT-HFE cells (**Figure 2A & B**). In addition, consistent with their higher cholesterol content, increased expression of HMG-CoA reductase (HMGCoAR) (a rate-limiting enzyme in cholesterol synthesis) and APOE (a protein involved in cholesterol transport) was also observed in C282Y-HFE cells (**Figure 2A & B**).

### Effect of exposing cells to inhibitors of cholesterol synthesis and transport on cell survival

**Figure 3:**
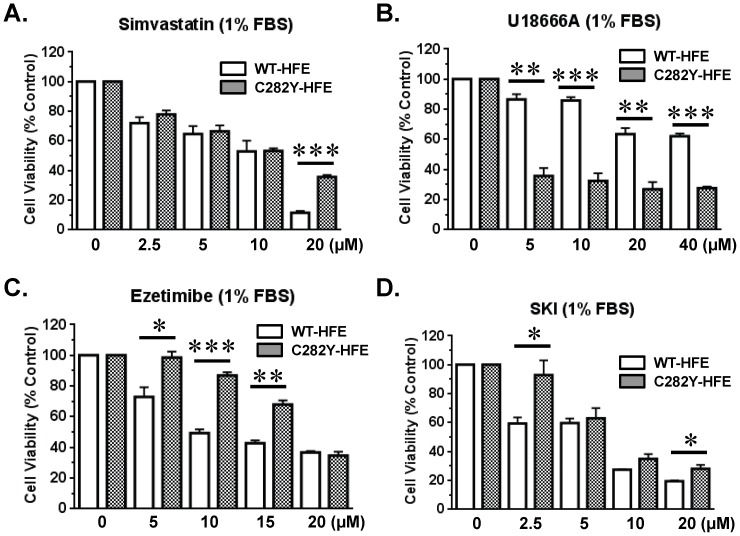
Effect of treatment of simvastatin, ezetimibe, U18666A, or sphingosine kinase inhibitor (SKI) on survival of WT-HFE and C282Y-HFE stably transfected human neuroblastoma cells. WT-HFE (2.4–3×10^4^/well of a 96 well plate) or C282Y-HFE (0.8–1.5×10^4^/well) stably transfected SH-SY5Y cells were cultured overnight in complete medium (10% FBS). After washing the cells with Hank’s buffer, they were exposed to different concentrations of either simvastatin (A), U18666A (B), ezetimibe (C), or SKI (D) in 1% FBS-containing medium and incubated at 37°C in an atmosphere of 5% CO_2_/95% air for 48 hr. At the end of that time, cell survival was measured using the MTS assay. Vehicle (1% DMSO) treated cells served as the 100% control. This is representative data of 3 different experiments. Data are displayed as means ± SEM. Some error bars are too small to be seen. The asterisk indicates a significant difference compared to controls. *p<0.05, **p<0.01, ***p<0.001 (n = 3).

To determine whether inhibition of cholesterol synthesis in C282Y-HFE cells enhanced cell death, we measured the effect of simvastatin, an inhibitor of HMGCoAR activity, on cell survival. After 48 hr of treatment 20 µM simvastatin was more effective at inhibiting viability of WT-HFE than C282Y-HFE expressing cells (**Figure 3A**). In contrast to results obtained using simvastatin, exposure of cells to U18666A, a cholesterol synthesis and transporter inhibitor, indicated that the C282Y-HFE cells were more sensitive to U18666A treatment than WT-HFE cells (**Figure 3B**).

The finding that C282Y-HFE cells had significantly higher expression of both the *NPC1L1* gene and protein than WT-HFE cells led us to interrogate the effect of ezetimibe, a NPC1L1 ligand that inhibits its ability to bind cholesterol, on cell viability. Treatment with low concentrations of ezetimibe for 48 hr resulted in a higher viability of C282Y-HFE cells compared to those expressing WT-HFE; use of 20 µM ezetimibe affected viability of both cell types similarly (**Figure 3C**).

### Effect of exposing cells to an inhibitor of sphingosine kinase on cell survival

Previously we reported that C282Y-HFE cells had significantly more sphingosine kinase 1 mRNA, and a concomitant increase in its protein expression and activity relative to WT-HFE cells [12]. A number of studies have shown an association between increased cell proliferation and cell migration and an elevation in sphingosine kinase 1 [14], [16], [17]. Therefore, we determined whether its inhibition would decrease survival of C282Y-HFE cells relative to cells expressing WT-HFE. While a significant dose-dependent reduction in survival of WT- and C282Y-HFE cells was observed relative to vehicle-treated controls, C282Y-HFE cells were less sensitive to treatment with SKI than WT-HFE controls (**Figure 3D**).

## Discussion

In this study, we found that C282Y-HFE neuroblastoma cells have increased cholesterol compared to WT-HFE neuroblastoma cells. The significant increase in expression of a number of genes involved in cholesterol metabolism and the concomitant increase in the proteins encoded by them provides a potential explanation for the observed increase in cell cholesterol in C282Y-HFE cells. The few published studies that describe the effect of C282Y-HFE expression on cholesterol indicate that hemochromatosis patients homozygous for C282Y-HFE have lower serum cholesterol and low-density lipoprotein (LDL) levels than normal controls [18]. Atherosclerosis patients homozygous for C282Y-HFE also have lower LDL levels than WT-HFE controls [19]. These are the first studies to investigate whether there is an association between expression of C282Y-HFE and cellular cholesterol in humans. The observation that cholesterol was increased in the C282Y-HFE cells relative to WT-HFE controls coupled with reports that statins might be used, to inhibit tumor cell growth, metastasis of tumor cells, and induction of apoptosis [20] supports the hypothesis that the overexpression of cholesterol in C282Y-HFE cells may be part of the explanation for its association with an increased risk of cancer. The increase in C282Y-HFE cell expression of genes/proteins involved in cholesterol uptake and transport provides a possible explanation for the lower levels of serum and low-density lipoprotein concentrations seen in individuals homozygous for C282Y-HFE.

In the present study, we observed dramatic changes (Table 1) in expression of some of the genes involved in cholesterol metabolism in C282Y-HFE cells compared to WT-HFE cells. However, the degree of change in mRNA levels was greater than the degree of change in protein expression observed for the proteins analyzed (figure 2). The fact that we only saw an 18% increase in total cholesterol in C282Y-HFE cells relative to WT-HFE cells may be due in part to the fact that they were maintained in medium containing 10% fetal bovine serum which is known to contain cholesterol.

We have identified a number of genes, related to cholesterol metabolism, that are over-expressed in C282Y-HFE cells and confirmed that three of the encoded proteins are also overexpressed. Many of the affected genes function in the transport and uptake of cholesterol. A significantly up-regulated gene involved in cholesterol uptake by cells is *NPC1L1* (increased 333-fold). It encodes the Niemann-Pick C1-Like 1 (NPC1L1), a trans-membrane protein that functions in uptake of cholesterol via clathrin-mediated endocytosis and whose regulation by cellular cholesterol content [21]-[24] appears to be disrupted by expression of C282Y-HFE. Another example of an affected transport protein is provided by the elevated expression of *CXCL16* (increased 306-fold), which encodes CXCL16/SR-PSOX, an interferon-γ–regulated chemokine found in both trans-membrane and soluble forms, and can function as a scavenger for oxidized low-density lipoproteins [25]-[27]. *LRP1B* (increased 198-fold) encodes the low density lipoprotein receptor related protein 1B (LRP1B). It interacts with the cytoplasmic tail of the LDL receptor and mediates cholesterol uptake [28]. *COLEC12* (increased 177-fold) encodes a member of the C-lectin family of proteins. This protein is known to function as a scavenger receptor and can recognize oxidized phospholipids and lipoproteins. *APOD* (increased 161-fold) encodes apolipoprotein D (APOD) which is associated with high density lipoproteins and interacts with lecithin:cholesterol acyltransferase during lipoprotein metabolism [29]. *APOE* (increased 71-fold) encodes apolipoprotein E (APOE), which is a ligand for LDL receptors as well as chylomicron remnant receptors and plays a key role in lipid transport from serum to cells as well as between cells in the central nervous system [30]-[32]. Collectively, these changes, indicate disruption of cholesterol homeostasis, particularly its uptake, and may account for the elevated cholesterol content found in C282Y-HFE expressing cells.

A number of the changes seen in gene expression support findings reported by other researchers. For example, elevated CXCL16 expression was observed in several prostate cancer cell lines, as well as in prostate tumors from humans, with much higher expression found in the more aggressive PC3 cells relative to the less aggressive LNCaP cells [33]. We found significant up-regulation of mRNA and protein expression of CXCL16 in C282Y-HFE neuroblastoma cells. These data suggest that the significant elevation of CXCL16 may in part contribute to the aggressive nature of C282Y-HFE cancers. The role of NPC1L1 in cancer has not received much attention. Given the role of cholesterol in cancer and the function of NPC1L1 in cholesterol uptake, future studies focusing on this connection may provide additional targets to inhibit cholesterol-dependent cancer progression.

As indicated previously, individuals that express C282Y-HFE have an increased risk of breast, colorectal, ovarian and prostate cancer relative to WT-HFE carriers [6]-[9]. It is possible that the increased risk is due in part to the C282Y-HFE induced alterations in lipid metabolism. In previous work we found that C282Y-HFE cells expressed significantly more S1P (cell growth promoter) and significantly less ganglioside GM1 (lipid raft component) than WT-HFE cells [12]. Here we have shown an elevation in the cholesterol content of C282Y-HFE cells. Cholesterol is an essential membrane component that plays an important role in maintaining membrane integrity and fluidity [34]. It is also critical for formation of lipid rafts where it serves as a spacer between hydrocarbon chains of sphingolipids [35], [36]. Therefore, it has been hypothesized that alterations in the cholesterol content of cells can change the properties of lipid rafts. In fact, studies have shown that depletion of cholesterol from the plasma membrane causes movement of raft-associated proteins into non-raft areas of the membrane affecting protein function and in turn, cell survival [36], [37]. The finding that C282Y-HFE, unlike WT-HFE, is not localized in lipid rafts [12], does not contribute to the regulation of iron uptake, and is responsible for elevated cellular cholesterol, provide possible explanations for why it is an increased risk factor for certain cancers.

Although conflicting data have been found for the effect of statin treatment on cancer risk [38], several reports have documented their efficacy as a treatment. For example, studies of patients on long-term statin therapy showed that they had a reduction in the incidence of prostate cancer [39]-[41], suggesting that lower cholesterol levels were protective. In addition, depletion of cholesterol from human prostate cancer cells harboring high amounts of cholesterol resulted in increased sensitivity to apoptosis [42]. Cholesterol depletion using methyl beta cyclodextrin (MβCD) activated cell death pathways and inactivated AKT, effects that were reversed by cholesterol repletion [42]. Similar effects were achieved by treating cancer cells with simvastatin [42] to inhibit HMGCoAR, the rate limiting enzyme in cholesterol synthesis. Although the level of cholesterol in human neuroblastomas is unknown, the results indicate that treatment of cholesterol-enriched C282Y-HFE neuroblastoma cells with U18666A alone significantly decreased their survival relative to WT-HFE cells. This may reflect the fact that U18666A inhibits desmosterol Δ24-reductase, a step that only affects cholesterol synthesis, as well as cholesterol transport from late endosomes/lysosomes to the ER while not affecting cholesterol transport to the plasma membrane [43]. The increased sensitivity of C282Y-HFE cells relative to WT-HFE controls to U18666A, but not simvastatin or ezetimibe, also indicates the importance of desmosterol Δ24-reductase in the pathway of cholesterol synthesis and/or cholesterol transport to the ER that is targeted in C282Y-HFE neuroblastoma cells. While simvastatin inhibits cholesterol synthesis it also inhibits synthesis of other isoprene derived products which may account for the similarity in its effect on cell viability at lower concentrations and its more pronounced effect on WT-HFE cells, which have less cholesterol to begin with, at 20 µM. The enhanced effect of ezetimibe (inhibitor of cholesterol uptake) at low concentrations on WT-HFE cell viability may also reflect their reduced concentration of cholesterol relative to C282Y-HFE cells. When exposed to 20 µM of ezetimibe, viability of both cell types was reduced by ∼80%. The greater susceptibility of C282Y-HFE cells to U18666A indicates that they may be more susceptible than WT-HFE cells to a drug that specifically inhibits a commiting step in cholesterol synthesis and that U18666A might be considered as a potential therapeutic.

While this study did not look at the relationship between C282Y-HFE and iron homeostasis *per se* we previously reported that C282Y-HFE cells are significantly more sensitive to the iron chelator desferoxamine (DFO) and that they have higher levels of intracellular iron than WT-HFE cells [5]. Whether the association between expression of C282Y-HFE and cholesterol metabolism is mediated by the C282Y-HFE protein, elevated intracellular iron, or downstream effects of either one still needs to be interrogated.

In conclusion, we found that C282Y-HFE expressing human neuroblastoma cells have increased cholesterol levels as a result of increased biosynthesis and uptake compared to WT-HFE expressing controls. We also found that targeting cholesterol synthesis with U18666A decreases cell viability and thus may be a therapeutic option for C282Y-HFE neuroblastoma cells. Given that cholesterol is elevated in different types of tumors and is implicated in the induction of cell survival pathways [44] findings from our studies suggest that the increase in total cholesterol in C282Y-HFE neuroblastoma cells could be a therapeutic target for cancer patients with this genotype. Our findings, consistent with others, support a role for the C282Y-HFE variant in malignancy and argue for stratification of subjects by HFE genotype to assess disease susceptibility and to develop effective therapeutics.
